# Investigation into detection and treatment rates of hyperactive and hypoactive delirium in the ICU setting

**DOI:** 10.1186/cc10948

**Published:** 2012-03-20

**Authors:** S Kudsk-Iversen, J Wong, H Kingston, L Poole

**Affiliations:** 1 The Royal Liverpool University Hospital, Liverpool, UK

## Introduction

We aimed to investigate the link between the type of delirium (that is, hyperactive or hypoactive), its detection by the day staff and the subsequent treatment. The morbidity related to delirium is well known to critical care medical staff; however, some findings suggest insufficient and inconsistent recognition and management of delirium [1]. Hypoactive delirium, despite being more common in the ICU setting, often goes undetected and undertreated due to its withdrawn and drowsy presentation [2].

## Methods

A prospective cohort study over 8 weeks in a 25-bed ICU setting. Daily CAM-ICU assessments were done by three trained doctors. It was noted whether the ICU team had assessed the individual patient for delirium. If the patient was delirious, the team was informed and their management was noted. Eligible patients had to have a RASS score above -4 and be able to comply with the assessment. The Fisher's exact test was used to calculate statistical significance of detection and treatment.

## Results

A total of 139 patients were included, providing a total of 507 patient-days. On 32 occasions (6%) the patient assessed was found to be delirious. Twelve patients in ITU (19%) and nine in HDU (9%) were delirious at least once. Of the 32 cases of delirium, 53% were hyperactive. Seventy-six percent of the hyperactive and 27% of the hypoactive cases had been detected by the day team (*P = *0.0118). Once the delirium was recognised, 76% of the hyperactive and 20% of the hypoactive cases were started on targeted treatment (*P = *0.0038). See Tables 1 and 2.

**Table 1:** Hyperactive and hypoactive cases and their detection rate

	Number of hyperactive (%)	Number of hypoactive (%)
Detected	13 (76)	4 (27)
Not detected	4 (24)	11 (73)

**Table 2:** Hyperactive and hypoactive cases and their treatment rate

	Number of hyperactive (%)	Number of hypoactive (%)
Treated	13 (76)	3 (20)
Not treated	4 (24)	12 (80)

## Conclusion

Although the study had a higher rate of hyperactive delirium compared to otherwise available research, the findings confirmed that a significant proportion of hypoactive delirium goes undetected and remains largely undertreated.
